# Molecular, genetic and transcriptional evidence for a role of *VvAGL11 *in stenospermocarpic seedlessness in grapevine

**DOI:** 10.1186/1471-2229-11-57

**Published:** 2011-03-29

**Authors:** Nilo Mejía, Braulio Soto, Marcos Guerrero, Ximena Casanueva, Cléa Houel, María de los Ángeles Miccono, Rodrigo Ramos, Loïc Le Cunff, Jean-Michel Boursiquot, Patricio Hinrichsen, Anne-Françoise Adam-Blondon

**Affiliations:** 1 Biotechnology Unit, La Platina Experimental Station, INIA, Av. Santa Rosa 11610, 8831314, Santiago, Chile; 2 UMR INRA CNRS University of Evry on Plant Genomics, 2 rue Gaston Crémieux, BP 5708, 91057, Evry, France; 3 INRA - Montpellier SupAgro, UMR 1097, Equipe Diversité Génétique et Génomique Vigne, 2 place P. Viala, F-34060 Montpellier Cedex 1, France

## Abstract

**Background:**

Stenospermocarpy is a mechanism through which certain genotypes of *Vitis vinifera *L. such as Sultanina produce berries with seeds reduced in size. Stenospermocarpy has not yet been characterized at the molecular level.

**Results:**

Genetic and physical maps were integrated with the public genomic sequence of *Vitis vinifera *L. to improve QTL analysis for seedlessness and berry size in experimental progeny derived from a cross of two seedless genotypes. Major QTLs co-positioning for both traits on chromosome 18 defined a 92-kb confidence interval. Functional information from model species including *Vitis *suggested that *VvAGL11*, included in this confidence interval, might be the main positional candidate gene responsible for seed and berry development.

Characterization of *VvAGL11 *at the sequence level in the experimental progeny identified several SNPs and INDELs in both regulatory and coding regions. In association analyses performed over three seasons, these SNPs and INDELs explained up to 78% and 44% of the phenotypic variation in seed and berry weight, respectively. Moreover, genetic experiments indicated that the regulatory region has a larger effect on the phenotype than the coding region. Transcriptional analysis lent additional support to the putative role of *VvAGL11's *regulatory region, as its expression is abolished in seedless genotypes at key stages of seed development. These results transform *VvAGL11 *into a functional candidate gene for further analyses based on genetic transformation.

For breeding purposes, intragenic markers were tested individually for marker assisted selection, and the best markers were those closest to the transcription start site.

**Conclusion:**

We propose that *VvAGL11 *is the major functional candidate gene for seedlessness, and we provide experimental evidence suggesting that the seedless phenotype might be caused by variations in its promoter region. Current knowledge of the function of its orthologous genes, its expression profile in *Vitis *varieties and the strong association between its sequence variation and the degree of seedlessness together indicate that the D-lineage MADS-box gene *VvAGL11 *corresponds to the *Seed Development Inhibitor locus *described earlier as a major locus for seedlessness. These results provide new hypotheses for further investigations of the molecular mechanisms involved in seed and berry development.

## Background

*Vitis vinifera *L genomic resources, including both released genomic sequences [1,2], allow the characterization at molecular level of the biological function of genes involved in agronomically interesting traits [3-6]. Stenospermocarpic seedlessness [7], found in popular table grape varieties for fresh or dried consumption such as Sultanina (Thompson Seedless), is one of these traits. In stenospermocarpic berries, pollination and fertilization occur but both the seed coat and endosperm cease their normal development at early stages, leaving undeveloped seeds or seed traces [7,8].

Seed and berry size depend on genetic background, and they both segregate in experimental populations with a continuous distribution indicative of polygenic determinism [8-11]. To increase the chances of obtaining new seedless varieties, breeding programs commonly cross two seedless parental genotypes and progeny are obtained through embryo rescue assisted by *in vitro *tissue culture [12]. The progeny thus obtained (n < 200 in general) are used to investigate the genetic basis of grape seedlessness and berry size [4,9-11,13-17]. The most accepted model proposed that genetic inheritance of seedlessness in grapevine is based on the expression of three independent recessive genes under the control of a dominant regulator gene named *SDI *(*Seed Development Inhibitor*) [10,13,14,18]. This model was partly confirmed by several studies that all reported a major QTL for seedlessness co-localizing with *SDI *on linkage group (LG) 18. This major QTL explains 50% to 70% of the phenotypic variation of the trait [4,9,10,15,16]. Numerous other minor QTLs were found on different LGs, but they were not reproducible across different seasons and were not present in all crosses. Thus, the molecular characterization of the *SDI *locus is a key step toward understanding the molecular mechanisms underlying seedlessness.

In *Arabidopsis *and other model species, genes involved in flower, ovule, seed and fruit development have been isolated and characterized from loss of function mutants. Among them, the MADS-box family plays an important role [19]. Most of the MADS-box genes identified in *Arabidopsis *seem to have counterparts in grapevine [20]. In spite of grapevine particular features, characterized MADS-box genes expressed during the reproductive development might have the same role than their functionally characterized orthologues in model species [3]. Among these MADS-box genes, *VvAGL11 *(*VvMADS5 *[21], *VvAG3 *[20]) shows homology to the *STK*/*AGL11 *gene in *Arabidopsis *and is expressed in mature carpels, developing seeds and pre- and post *véraison *fruits; this expression suggests a possible role for this gene in ovule, seed and berry development in grapevine [21]. *VvAGL11 *was also mapped *in silico *to the same contig that contains the *SDI *locus and the closest marker to a seedlessness QTL (SSR VMC7F2 [4]), suggesting that it might play an important role in seed development. In parallel, a transcriptional analysis of genes differentially expressed in the flowers of seeded and seedless Sultanina lines allowed the identification of a chloroplast chaperonin (ch-Cpn21) whose silencing in tobacco and tomato resulted in seed abortion [22], and of a ubiquitin extension protein (S27a) having a probable general role in the control of organ development in grapevine [23]. None of these genes co-segregated with the *SDI *locus. Besides these works, no further evidence has been generated to unveil the genetic control of seedlessness in grapevine.

Genetic analyses have also revealed a major QTL for berry size [4,9,10,16] and ripening date [4,10,16] that overlap with the major seedlessness QTL on LG 18. The complex developmental process modified by genetic, physiological and environmental factors that underlies berry development was first reviewed by Coombe [24] and was very recently updated by Carmona et al. [3]. The relationship between seed number and berry size was reviewed by Ollat et al. [25]. These overlapping QTLs detected by genetic experiments could be reflective of pleiotropic effects caused by hormones in developing seeds [9,16]. However, most of the phenotypic variation for berry size is not explained by the *SDI *locus [9,10,16], and there is still room for the identification of other loci involved in seed and berry development. The molecular biology of fleshy fruit ripening has received considerable attention [26,27], but little is known about the determinants of early fleshy fruit morphogenesis. Differential screening of ESTs and berry transcriptomic analysis identified several genes that show differential expression during young fruit development, the onset of *véraison *and ripening [26,28-31].

In this work, we designed a strategy to test the hypothesis for a possible role of *VvAGL11 *in seeddlessness. We integrated multiple genomic resources as soon as they became available to contribute to the molecular characterization of the *SDI *locus: QTL mapping in seedless × seedless derived progeny [16], physical mapping on a Cabernet-Sauvignon physical map [5] and the released sequence of grapevine [1], which gave further positional evidence for *VvAGL11 *as being the major gene responsible for seedlessness [4]. Here, we provide genetic and transcriptional support for this hypothesis and discuss its potential for molecular-assisted breeding programs.

## Results

### Phenotypic evaluation

Phenotypic evaluations of plants grown in their own roots (2007 season) and over Sultanina rootstocks (2009 and 2010 seasons) confirmed the distribution of seed and berry weight previously reported by Mejía et al. [16] for the same progeny (Additional file 1). Neither of the two traits fit a normal distribution (*P*-value < 0.005) according to the Anderson-Darling normality test. Non-parametric Spearman analysis showed a correlation between mean seed fresh weight per berry (SFW) and mean berry weight (BW) of 69.0%, 67.8% and 64.6% for the 2007, 2009 and 2010 seasons, respectively (α = 0.05). However, variations in BW values were explained by a weak linear relationship with SFW (r^2 ^= 0.41, 0.43 and 0.46; *P*-value < 0.0001; F-value = 77.17, 98.35 and 106.70 for the 2007, 2009 and 2010 seasons, respectively Additional file 2).

Most of the heterozygous genotypes of the population, defined as such by the SSR VMC7F2 marker tightly linked to the *SDI *locus, were seedless and showed an average SFW below the population average, like (for instance) both heterozygous parental genotypes. The calculated dominance effect *d *was negative, showing that the seedless allele presents incomplete dominance (partial dominance) over the seeded allele. This partial dominance effect was also detected for berry weight, but the effect was lower. Finally, several offspring exhibited extreme phenotypes relative to the parents for both traits (Additional file 1). This phenotypic distribution was consistent with the heterozygosity in both parental genotypes of the *SDI *locus and the partial dominance of the seedless allele.

### Construction of linkage group 18

**Figure 1:**
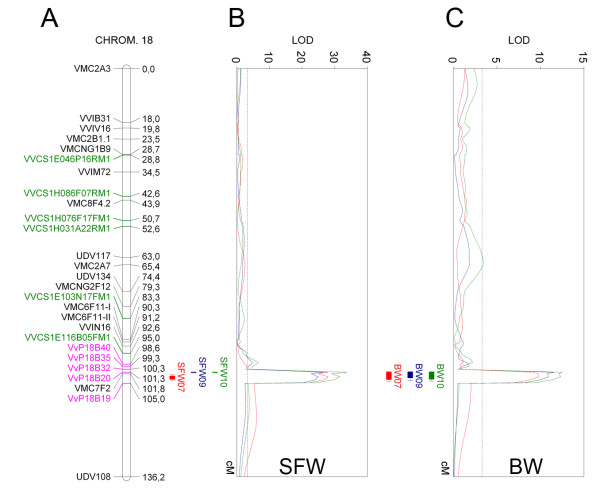
**Localization of the major QTLs for seedlessness and berry size detected over three different seasons on chromosome 18**. A: Consensus genetic map of chromosome 18 based on the RS × S progeny. Green and pink markers correspond to SSRs developed in this study from Cabernet Sauvignon BAC End Sequence and from contig assemblies of the grapevine genome sequencing project respectively. B and C: Projected seedlessness and berry size QTLs represented by colored vertical bars and LOD (logarithm of the odds) profiles to the right of chromosome 18. Red, blue and green lines correspond to 2007, 2009 and 2010 seasons, respectively. Bar lengths are representative of their confidence interval once projected on the consensus map. Seedlessness was analyzed as seed fresh weight (SFW) and berry size as berry weight (BW). 1-LOD and 2-LOD support intervals were used for the prediction of the confidence intervals. Vertical dashed line in the LOD profile represents the LOD threshold for significant QTLs according to the permutation tests. Genetic distances are expressed in centimorgans (cM).

Taking into account a former QTL detection experiment [16] and other results [4,9,10,15] that all showed the presence of a major QTL for seedlessness on LG 18, we replaced dominant markers and increased marker density with available and newly developed co-dominant markers. For this purpose, 15 new SSRs linked to the targeted regions were designed taking advantage of the available genomic resources (Cabernet Sauvignon BAC End Sequences (BES), or the Pinot Noir PN40024 6X genome assembly), and they were genotyped in the same experimental population. As an example, the microsatellite VMC7F2, previously reported as the nearest marker to the *SDI *locus [18] and the closest marker to the peak of the major QTL for seedlessness and berry size [9,16], was localized on the Cabernet-Sauvignon physical map on BAC contig_1821. BES from this BAC contig were searched against the 6X genomic assembly of the grapevine genome. Five SSRs (VvP18B40, VvP18B35, VvP18B32, VvP18B20 and VvP18B19) identified in these genomic sequences could be mapped (genetically, physically and *in silico*) to the vicinity of VMC7F2 (Figure 1A). With this strategy, only 11 new markers were consistently positioned on both parental linkage maps (Additional file 3). The mapping data set for LG 18 in Ruby Seedless (RS) and Sultanina (S) included a total of 27 co-dominant markers (Additional file 4), among which were six BES-derived SSRs and five genomic assembly-derived SSRs. The consensus linkage map built with these data covers 136.2 cM with a mean inter-locus distance of 5 cM (Figure 1A). No significant differences in distances or positions were observed between the two parental maps (not shown).

### Seedlessness and berry weight QTL analysis

Improvements that were made based upon a former study [16] (expansion of the phenotyped population from 85 to 115, 126 and 122 genotypes in the 2007, 2009 and 2010 seasons, respectively, an increase in the number of berries sampled for phenotypic evaluation and an improved genotyping strategy) resulted in more accurate QTL detection. A narrower (down to 1.5 cM for SFW and 4.5 cM for BW, Table 1) and more reliable confidence interval (based on co-dominant markers) was established for the major QTL identified on LG 18 for seed and berry size (Figure 1B and 1C, and Table 1).

**Table 1:** QTLs identified for seed fresh weight (SFW) and berry weight (BW) on the consensus linkage group 18

Trait	Season	Closest Marker to LOD peak	LOD	CI (cM)	Var. Expl. MQM (%)	Marker Highest K-W	Var. Expl. K-W (%)	P (K-W)	Mean (g) class: aa	Mean (g) class: ab	Mean (g) class. bb
		**Without intragenic markers for VvAGL11**									
		
**SFW**	**2007**	VMC7F2	28.0	1.5	61.7	VMC7F2	75.7	0.0001	0.003	0.009	0.062
	**2009**	VvP18B20	26.5	1.5	61.5	VMC7F2	67.7	0.0001	0.009	0.016	0.078
	**2010**	VvP18B20	33.8	1.5	71.2	VMC7F2	78.8	0.0001	0.005	0.009	0.061

**BW**	**2007**	VMC7F2	9.8	3.5	33.0	VMC7F2	38.5	0.0001	1.239	1.682	2.457
	**2009**	VvP18B20	12.0	3.5	33.9	VMC7F2	40.1	0.0001	2.061	2.436	3.670
	**2010**	VvP18B20	12.0	3.5	36.1	VMC7F2	42.5	0.0001	1.512	1.877	2.891

		**With intragenic markers for VvAGL11**									
		
**SFW**	**2007**	p3_VvAGL11	24.0	0.6	61.4	VMC7F2	75.7	0.0001	0.003	0.009	0.062
	**2009**	p3_VvAGL11	26.3	0.6	61.2	p3_VvAGL11	69.8	0.0001	0.007	0.017	0.080
	**2010**	p3_VvAGL11	32.2	0.6	69.5	VMC7F2	78.8	0.0001	0.005	0.009	0.061

**BW**	**2007**	p3_VvAGL11	9.2	0.9	31.1	VMC7F2	38.5	0.0001	1.239	1.682	2.457
	**2009**	p3_VvAGL11	10.8	0.6	32.3	VMC7F2	40.1	0.0001	2.061	2.436	3.670
	**2010**	p3_VvAGL11	14.7	0.6	41.8	p3_VvAGL11	44.4	0.0001	1.390	1.855	2.886

For both traits, the QTL analysis was performed over three different seasons with and without intragenic *VvAGL11 *markers. The table shows the closest marker to the peak in the LOD profile, the LOD value for the same marker (LOD), the 1-LOD support confidence interval (CI), the proportion of phenotypic variance explained by QTLs with parametric and non-parametric analysis (Var. Expl. MQM and Var. Expl. K-W respectively), the P-value for the Kruskal-Wallis test (P), and the mean seed fresh weight or berry weight values for genotypic classes (*aa*, *ab and bb*,) detected in the RS × S progeny.

Parametric QTL analyses (IM and MQM) did not reveal significant differences between the parental genotypes in any of the evaluated seasons (2007, 2009, and 2010) for either of the two analyzed traits (not shown). Co-localizing QTLs were detected for SFW and BW, both centered on the VMC7F2 marker that was used as a cofactor for MQM analysis (Figure 1B and 1C). These QTLs explained most of the phenotypic variation in SFW (67.1%, 61.5% and 71.2% for the 2007, 2009 and 2010 seasons, respectively), and a minor proportion of the phenotypic variation in BW (33.0%, 33.9% and 36.9% for the same seasons, respectively; Table 1). Non-parametric analysis performed with the same marker used as a cofactor in the MQM analysis (VMC7F2) gave the highest Kruskal-Wallis values for SFW (75.7, 67.7 and 78.8 for the 2007, 2009 and 2010 seasons, respectively) and BW (38.5, 40.1 and 42.5 for the same seasons). Other minor QTLs were found on other linkage groups. However, none of them were consistent across seasons or in previous analyses performed in the same or other progeny [4,9,10,15,16]. Therefore, these other minor QTLs were not further assessed in the present work.

### Positional candidate gene identification for SFW and BW

**Figure 2:**
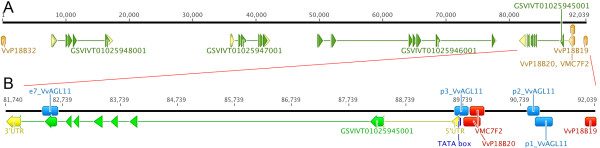
**Structure of putative candidate genes identified in the Confidence Interval of both major QTLs for seedlessness and berry size**. A: Confidence Interval defined by newly developed SSRs VvP18B19 and VvP18B32 anchored on the 12 × genome assembly for both seedlessness and berry size co-positioning QTLs. Positional candidate gene models were directly imported from the Grape Genome Browser except for GSVIVT01025945001 that was manually curated. Yellow and green segments denote UTRs and exons respectively. Orange segments outside the sequence correspond to genetically mapped SSRs in the RS × S progeny. B: Detailed structure of the most probable candidate gene, *VvAGL11 *(GSVIVT01025945001). Yellow, green and blue segments represent UTRs, exons and the TATA-box respectively. Red and light blue segments correspond to mapped SSRs developed from genomic resources (except VMC7F2) and intragenic markers developed from allele sequencing, respectively.

Of the two co-localizing QTLs for BW and SFW, BW defined the largest confidence interval (CI), which was flanked by SSR markers VvP18B19 and VvP18B32, defining a region equivalent to ~92 kb (chr18:26806909..26898947 [32]) in the 12x genome assembly of Pinot Noir PN40024. This region contains four gene models (Figure 2A and Additional file 5) confirmed by alignments with *Vitis vinifera *cDNAs from public databases. As expected, among these gene models, GSVIVT01025948001 (Embl:CAO16376) is an ortholog of the *AGAMOUS-like 11 *gene of *Arabidopsis *(*AGL11 *[33,34]), with 75% amino acid identity (10% above other described orthologs, not shown) and 86% positive matches (Figure 2B). *AGL11 *belongs to the D-lineage MADS box family responsible for ovule identity in monocotyledons and dicotyledons [34,35]. The protein alignment of the C and D lineages of the AGAMOUS family from different plant families and the construction of a phylogram showed that these lineages evolved from a common ancestor during angiosperm evolution [36] (Additional file 6). The alignment also indicated that *VvMADS5*, isolated and characterized in cv. Syrah [21], is likely to be allelic (99.1% amino acid identity) to the *VvAGL11 *sequences obtained from Sultanina (Additional file 6).

Lacking evidence that any of the remaining three annotated genes from this region could be involved in seed or berry development (Additional file 5), we decided to concentrate our further analysis on *VvAGL11*. Indeed, in grapevine, *VvAGL11 *has been shown to have carpel-specific RNA expression and to be highly expressed in flowers after the cap has been shed and in seeds [20,21]. All these results and current knowledge of the possible functions of the genes in the region confirmed the former hypothesis of Costantini et al. [4] that *VvAGL11 *is the best positional candidate gene for the control of seed development. To obtain more evidence for a possible role of *VvAGL11 *in seedless table grapes, this positional candidate gene was characterized at the molecular, genetic and transcriptional levels.

### Molecular characterization of *VvAGL11 *alleles

Based on their genotype at the VMC7F2 marker, both Ruby Seedless and Sultanina are heterozygous in the *VvAGL11 *region (Table 2). *VvAGL11 *sequences (regulatory and coding) were thus isolated from homozygous genotypes showing a stable seeded or seedless phenotype among the RS × S progeny. As Ruby Seedless inherited the seedless allele from Sultanina, the isolated seedless allele was called an indifferently seedless allele whatever its origin (Sultanina or Ruby Seedless). The seeded allele from Sultanina, Ruby Seedless or Pinot Noir (PN40024) was called indifferently seeded allele.

**Table 2:** Genotype, phenotype and relative expression of *VvAGL11 *of stable seedless or seeded individuals

	Ruby Seedless	Sultanina	109	159	108	146	Red Globe
	
Origin	Emperor × (Muscat of Alexandria × Sultanina)	natural	RS × S	RS × S	RS × S	RS × S	(Hunisia × Emperor) × ((Hunisia × Emperor) × Nocera)
**Genotype**							
VMC7F2	ab	ab	aa	aa	bb	bb	bc
p3_VvAGL11	ab	ab	aa	aa	bb	bc	bb
e7_VvAGL11	ef	eg	ee	ee	fg	fg	fg

**Phenotype**							
SFW	0.0132	0.0088	0.0010	0.0021	0.0081	0.0419	0.1645
Relative SFW	13.2	8.8	1.0	2.1	80.5	41.9	164.5

**VvAGL11 expression**							
Normalized transcript abundance	0.001828	0.002582	0.000224	0.000223	0.006185	0.005227	0.006895
Relative expression	8.2	11.6	1.0	1.0	27.8	23.5	31.0

The pedigree of each analysed genotype is indicated. Mean seed fresh weight/berry (SFW), SFW relative to the minimum value, the normalised expression of *VvAGL11 *in berries at pea stage and the expression of *VvAGL11 *relative to the minimum value.

#### Sequence polymorphisms in the promoter region and in putative regulatory elements

In the reference genome PN40024 [1], *VvAGL11*'s putative regulatory region is defined as ~1,600 bp upstream of the TATA box and by a 5'UTR disrupted by an intron of ~ 1,200 bp (Figure 2B). Flanked by the same 5' and 3' ends, the seeded and seedless regulatory regions are 2,794 and 2,823 bp long, respectively. PN40024 and the seeded allele share 99.7% identity. By contrast, the seeded and seedless regulatory sequences have 96.8% identity with 13 INDELs and 22 SNPs differentiating the two alleles.

47 out of 118 cis-regulatory elements identified by PLACE [37] vary in number and position (Additional files 7 and 8). Among them several (GAGA)n cis-regulatory elements were identified as polymorphic in the putative regulatory region of *VvAGL11 *upstream and downstream from the transcription start site. In the Cauliflower Mosaic Virus 35S gene, GA-rich motifs positively affect promoter activity even when translocated upstream of the transcription start site [38], and in *Arabidopsis*, the first intron of *AGL11 *contains GA-rich motifs required for ovule- and septum-specific expression [39]. Thus, the putative cis-regulatory elements identified in the 5'UTR intron of *VvAGL11 *might be functional. The SSR markers VMC7F2 (consistently reported as the closest marker to the *SDI locus *[4,9,15,16]) and VvP18B20 (reported in this work) are located 420 and 350 bp, respectively, upstream of the TATA-box of the *VvAGL11 *gene, and the polymorphisms revealed by these SSR are (GAGA)n repeats (Additional file 8).

#### Sequence polymorphisms in the coding sequence

**Figure 3:**
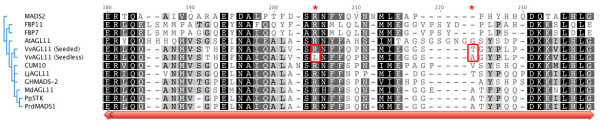
**Alignment of the conserved C-domain of plant D-lineage MADS-box proteins including both Sultanina-derived seeded and seedless alleles**. The Jukes-Cantor model was used for determination of genetic distance and the tree was built with UPGMA. Sequences have the following origin: *Lilium longiflorum*, *MADS2 *[GenBank:AAS01766]; Petunia hybrida, *FBP11 *[GenBank:CAA57445]; *Petunia hybrida, FBP7 *[GenBank:CAA57311]; *Arabidopsis thaliana, AGL11 *[GenBank:NP_192734]; Sultanina Seedless and Seeded-derived alleles of *VvAGL11; Cucumis sativus, CUM *[GenBank:AAC08529]; *Lotus japonicus, LjAGL11*, [GenBank:AAX13306]; *Gossypium hirsutum, GHMADS-2*, [GenBank:AAN15183]; *Malus × domestica, MdAGL11*, [GenBank:CAA04324]; *Prunus persica, PpSTK*, [GenBank:ABQ85556]; and *Prunus dulcis, PrdMADS1*, [GenBank:AAY30856]. Amino acidic differences between grapevine seeded and seedless alleles are indicated by red boxes and asterisks.

The CDS region of *VvAGL11 *was 100% identical between the seeded alleles isolated from the homozygous seeded individual and the predicted cDNA sequence from Pinot Noir (PN40024), whereas eight SNPs were identified between the seeded and seedless alleles (99% identity). Six of them were located in exon 7, two causing non-silent mutations (nt 590 C > T and 628 A > G; aa 197 R > L and 210 T > A; Additional file 9). The characterization of the progeny by SSCP marker e7_VvAGL11 (Figure 2B) later revealed the existence of a second seeded allele segregating in the RS × S progeny. e7_VvAGL11 alleles were thus amplified and sequenced from the different genotypic classes identified in the RS × S progeny: *ee*, *ef*, *eg*, *fg; *where *e *denotes the seedless allele. Seeded *f *and *g *alleles differed by one SNP in exon 7 that produced a silent mutation (Additional file 10). The C-domain, encoded in part by exon 7, is the less conserved domain within this gene family [40] (Figure 3). The R > L mutation, detected only in the seedless Sultanina-derived allele, affects one of the conserved motifs, and in *Arabidopsis *it has been shown that this C-terminal region might be a transactivation domain or contribute to the formation of multimeric MADS-box protein complexes [40-42]. To check for a possible association between the R > L mutation and the seedless trait, exon 7 was sequenced in a collection of 21 individuals: one wild *Vitis vinifera *genotype, five representatives of other species of the *Vitis *genus and fifteen cultivated *Vitis vinifera*, among which were one additional seedless variety (Kichmich noir), eight seeded table varieties and seven wine varieties. No additional SNPs or INDELs other than those identified in the RS × S background were found in this exon in the whole set of genotypes, although they were arranged into six haplotypes instead of the three segregating in the RS × S population (Additional file 10). The most frequent haplotype was the seeded allele found in Sultanina (the g allele, Additional file 10**)**. It seems to be conserved across the genus with nearly no variation observed at the interspecific level (Additional file 10). A T > A non-silent mutation was found in five table grapes (including Kichmich Noir, Sultanina and Ruby Seedless) that are seedless and one wine variety (Assyl Kara). The R > L mutation was observed in the seedless varieties (in the heterozygous state) but also in the seeded variety Assyl Kara (in the homozygous state) (Additional file 10). These results suggest that this mutation does not by itself explain the seedless phenotype.

### Genetic characterization of *VvAGL11 *alleles

To acquire more precise information about a possible role of the coding and/or putative regulatory region of *VvAGL11 *in the seeded versus seedless phenotype, intragenic markers derived from allele sequencing were designed to perform a QTL analysis. Markers p1, p2 and p3_VvAGL11 were designed to genetically analyze INDELs in the regulatory region (Figure 2B and Additional file 3). An INDEL revealed by p1_VvAGL11 affects a putative O2-like box, p2_VvAGL11 marks a putative TATA-box near far the transcription start site and p3_VvAGL11 marks a (GAGA)n motif. Finally, marker e7_VvAGL11 was designed to test SNPs identified in exon 7 (Figure 2B, Additional file 7 and Additional file 3).

**Figure 4:**
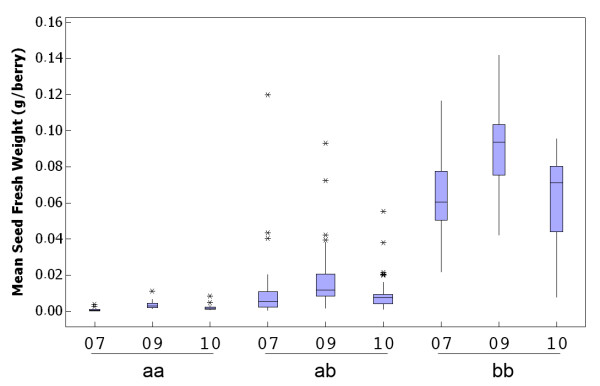
**Seed fresh weight depends on the specific combination of *VvAGL11 *alleles**. Intragenic marker p3VvAGL11, located in the regulatory region nearby the TATA box of candidate gene *VvAGL11*, explains the largest proportion of phenotypic variation in the experimental progeny RS × S and has a 1:2:1 (ab × ab) segregation where "a" and "b" stand for the seedless and seeded allele, respectively. The Box Plot shows the stratification of the experimental population using p3VvAGL11 that classifies the experimental population in three genotypes (two homozygous genotypes, "aa" and "bb", and one heterozygous "ab"). Also, the partial dominance effect of the seedless allele over its seeded counterpart is noticeable since heterozygous genotypes do not have an intermediate seed fresh weight. Outliers are represented by asterisks. Sample sizes were N = 115, 126 and 122 genotypes for 2007 (07), 2009 (09) and 2010 (10) seasons, respectively. Box width is proportional to the number of genotypes under each group.

Genetic mapping with intragenic markers reduced the SFW and BW QTL confidence intervals down to 0.6 and 0.8 cM, respectively (Additional file 11). The Kruskal-Wallis non-parametric method for QTL analysis was used to test the efficiency of these markers in the RS × S population. For all three analyzed seasons, the markers showing the highest correlation with seedlessness were VMC7F2 and p3_VvAGL11 (K = 75.7%, 67.7% and 78.8% for VMC7F2 in seasons 2007, 2009 and 2010, respectively; and K = 73.3%, 69.8% and 78.3% for p3_VvAGL11 in the same seasons, *P *< 0.0001; Table 1). A similar pattern was observed for berry weight, but with K values explaining 38% to 44% of the phenotypic variation (Table 1). A strong correlation was also found for both traits with *p1_VvAGl11*, p2_VvAGL11 and e7_VvAGL11; however, p3_VvAGL11 (which segregates 1:2:1 (*ab *× *ab*)) was found to be the best marker in terms genotypic and phenotypic association across the three evaluated seasons, as no false positives or negatives were identified in the homozygous genotypes (*aa*) or (*bb*) (Figure 4). This genetic evidence shows that the region delimited by marker VMC7F2 and the TATA-box (containing marker p3_VvAGL11) makes the largest contribution to the seedless phenotype in the Sultanina genetic background, suggesting that this region (~ 430 bp) might contain the causative genetic variation of the seedless phenotype. The stratification of the progeny by genotype (*aa*:*ab*:*bb*; Figure 4) defined by the p3_VvAGL11 marker (1:2:1) revealed a partial dominant effect of the seedless allele (*a*) over the seeded allele (*b*), which is consistent with the dominance effect observed at the phenotypic level only. This incomplete dominance effect is also observed for berry weight but with a minor effect (Not shown).

### Transcriptional characterization of *VvAGL11 *alleles

**Figure 5:**
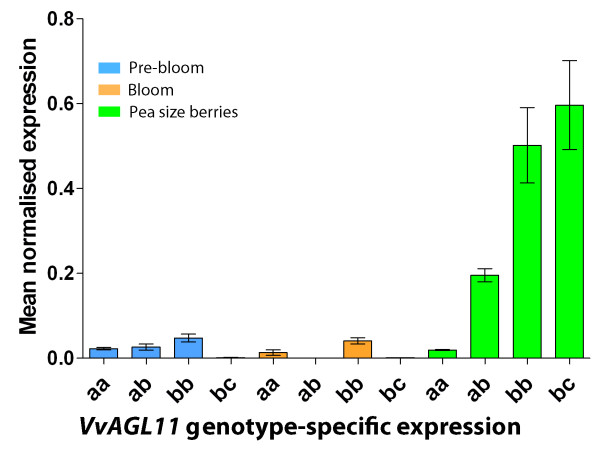
***VvAGL11 *transcript profile is genotype dependent at key stages of seed development**. The candidate gene *VvAGL11 *is expressed preferentially at pea size berry development stage and in seeded genotypes ("bb" and "bc"). Homozygous genotypes for the seedless allele ("aa") have a basal expression level, and as expected, heterozygous genotypes ("ab") have an intermediate level of expression. Candidate gene transcript relative abundance was quantified by qPCR along three key stages of seed and berry development in four genotypes differing on their degree of seed development (Table 2). Development stages are pre-bloom (light blue bars), bloom (orange bars) and pea size berries (light green bars). Genotypes for qPCR analysis were chosen among the experimental progeny RS × S based on their genotype defined by intragenic marker VMC7F2 that has a 1:2:1 (ab × ab) segregation where "a" and "b" stand for the seedless and seeded allele respectively. Additionally Red Globe, a seeded table grape variety, was also included ("bc" genotype). Each bar of the analysis represents the average expression between biological replicates. The expression of *VvAGL11 *was normalized towards EF1-α in the corresponding samples and the results are presented as percentage of the highest value of relative abundance.

Expression of *VvAGL11 *was analyzed by real-time PCR analysis at three key developmental stages for ovule and seed development: pre-bloom, bloom and pea-size berries. The samples came from seven genotypes: two seedless and two seeded homozygous seedlings of the RS × S progeny, both seedless heterozygous parental genotypes (RS and S) and a common seeded table grape genotype that contains two different seeded alleles: Red Globe (Table 2). In the seeded genotypes, *VvAGL11 *gene was expressed after anthesis, while in pre-bloom and bloom stages expression remained minimal. During the pea-size stage, its expression was 25 times higher than in pre-bloom or bloom stages (Figure 5), which is consistent with previous results [20,21]. Within the pea stage of development, the level of *VvAGL11 *expression was associated with the *VvAGL11 *genotype (Figure 5 and Table 2): genotypes homozygous for the seeded allele showed transcription 25 times higher than genotypes homozygous for the seedless allele, and the basal level was detected at earlier developmental stages. As expected, heterozygous genotypes showed an intermediate level of expression (Figure 5 and Table 2). All these differences were statistically significant, whereas no statistically significant difference in *VvAGL11 *expression in pea-stage berries was observed between the *bb *and *bc *seeded genotypes.

### Validation of intragenic VvAGL11 markers in different genetic backgrounds

To extend the genetic analyses performed in the experimental progeny (RS × S) to different genetic backgrounds, an association analysis was performed with a population of 146 genotypes characterized quantitatively for seed fresh weight. The population, derived mainly from crosses of ten seedless varieties, revealed p3_VvAGL11 as the marker that explains the largest proportion of phenotypic variation. For markers VvP18B19, VMC7F2, p1, p2, p3_VvAGL111 and VvP18B32, the statistic Kruskal-Wallis values were 53.3, 56.0, 60.4, 63.8, 66.3 and 52.1 (*P *< 0.0001), respectively.

The p3_VvAGL11 marker revealed six different alleles (176, 188, 190, 192, 196 and 198 bp) and seven main genotypes (four additional at very low frequency). Most of the genotypes harboring one or two copies of the 198-bp allele have a seedless phenotype (Additional file 12). As described for the experimental progeny (198 and 188 bp alleles), the seedless allele (198 bp) has partial dominance over the 188 and 192 bp seeded alleles; however, the same effect was not detected with respect to the 176 bp seeded allele. Interestingly, all of the genotyped seedless varieties within this analysis were heterozygous for this locus (not shown).

## Discussion

### Genetic dissection of seedlessness

Major QTLs for seed and berry weight were previously detected on LG18 in a subset of this progeny [16], in progeny derived from two other partially seedless genotypes [10] and in progeny derived from a cross of seeded and seedless genotypes [9]. For SFW, confidence intervals varied between 6 and 12 cM in Doligez et al. [10], 6 and 8 cM in Cabezas et al. [9] and 20 cM in Mejía et al. [16]. In the present work, integration of all the available genomic resources allowed us to quickly develop new co-dominant markers in the targeted area and to further reduce the confidence interval for this trait down to 1.5 cM with a segregating population of only ~ 125 phenotyped individuals. As the development of a well-balanced population in terms of phenotypic classes for seedlessness requires a step of *in vitro *embryo rescue [14], any strategy aiming to increase the accuracy of QTL detection without increasing the population size is of great interest. Moreover, genetic mapping of intragenic *VvAGL11 *markers, in addition to revealing a putative functional role of the regulatory of the coding region of *VvAGL11*, resulted in a narrower confidence interval (0.6 cM) for the SFW QTL, so far the narrowest QTL identified for this trait.

According to the genetic size of the most comprehensive SSR-based map for *Vitis vinifera *L. [43] and to the genome size reported for the grapevine genome [1], a confidence interval of 1.5 cM should be equivalent to ~ 500 kb. In our study, the confidence interval is equivalent to ~92 kb, indicating that this region may be hot spot for recombination, which allowed the mapping of intragenic *VvAGL11 *markers in a small progeny set (Additional file 13). However, genotyping errors in data sets are the most common source of variation and inflated genetic distances [44,45]. For instance, intragenic variation could be due to replication slippage [46], the mutation mechanism that cause the hypervariability of microsatellites ([47,48] cited in [49]). The putative regulatory region of *VvAGL11 *contains at least nine intragenic microsatellites annotated as (GAGA)n boxes (Not shown) with repeat units that vary from 4 to 13. Two genotypes of the RS × S experimental progeny presented a mutation, identified by SSR genotyping and sequence-verified, in the region amplified by marker p3_VvAGL11 (data not shown). This mutation consists of one additional unit of the GA repeat, which could have arisen either by Taq polymerase slippage during PCR or by a real mutation occurring in these genotypes. The use of a proofreading polymerase for the amplification and sequencing supports the latter hypothesis (data not shown). The limited size of our experimental population is also a potential source of distortions in genetic distance and QTL effect estimations. It is now well known that in such small populations, major effect QTLs are detected properly, but mapping experiments should be refined with larger populations and/or experimental designs adapted for the detection of environmental effects and minor QTLs [50,51]. Indeed, the minor QTLs for BW and SFW detected in the present study, detected earlier in the same experimental population [16] and detected in other studies are neither coincident with each other nor stable among years [4,9,10,15]. The positive correlation of seed and berry weights and the co-localization of major QTLs for both traits observed in this study was also detected and described in other progeny sets [9,10,15]. As already discussed in these former papers, this correlation could be due to (i) one underlying gene having a direct effect over ovule and seed development and indirectly affecting berry development through growth regulators produced by the developing seed, (ii) one or several genes having different and independent impacts on seed and berry development, or (iii) to a combination of both alternatives. The argument in favor of a pleiotropic effect of one gene is based on the fact that the growth of fleshy fruits mainly relies on cell division at early stages of berry development but on cell expansion after *véraison *[52]. Cell division and expansion are both controlled by gibberellins, cytokinins and auxins, which are imported from seeds or ovules [53]. However, because the partial dominance effect observed for seedlessness was less pronounced with respect to berry weight, it is probable that the same or other underlying genes have an independent influence on berry development.

### Molecular dissection of the major QTL for seedlessness

The reduced confidence interval for the major seedlessness QTL corresponds to a 92 kb region of the grapevine genome sequence that contains four gene models. One of these corresponds to an ortholog of the MADS-box gene *AGL11 *in *Arabidopsis thaliana *[34] and *FBP11 *in *Petunia hybrida *[35], which were shown to be involved in the control of ovule identity. Based on current knowledge, none of the other genes are candidates for seed or berry development. Moreover, earlier expression studies in grapevine [21] suggested that *VvAGL11 *might influence ovule and seed development and that an alteration in this gene could yield seedless grapes. The expression profile described in this work for the seeded allele of *VvAGL11 *is consistent with what had been already reported in Syrah [21] and in Tempranillo [20], but also with the expression of orthologous genes like *AGL11 *[34,39,54], *FBP11 *[35,55], *TAGL11 *[56] and *OsMADS13 *[57]. In *Arabidopsis*, Pynyopich et al. [54] showed that *AGL11 *is strongly expressed in the funiculus starting from the initial stages of ovule development, in mature ovules and after fertilization. They also showed that in *agl11 *mutants, seeds are rounder and smaller than in the wild type, and that funicular cells are greater in number and size, indicating that *AGL11 *is also required to prevent abnormal growth of the funiculus. Among the MADS-box genes known to control ovule identity [33,54], *AGL11 *is the only one that seems to be both necessary and sufficient to promote ovule development [54]. The others have proven to be redundant, suggesting that some of them evolved from a common ancestral gene [54]. In *Arabidopsis*, the ectopic expression of *STK *(*AGL11*) promotes carpel development [33], and in grapes *VvAGL11 *is highly expressed in carpels [21] which ultimately develop into fruit, supporting the hypothesis that *VvAGL11 *might have a direct influence on berry development instead of merely a pleiotropic effect through seed development.

Alignment of the *VvAGL11 *and *AGL11 *nucleic and protein sequences showed that although the two proteins share 75% amino acid identity, no significant similarity exists between their promoter sequences. However, both predicted promoters are similar in length (~ 2.8 kb) and share 68% (93 of 136) of their cis-regulatory elements according to a signal scan performed with the PLACE database [37] over the *AGL11 *and *VvAGL11 *(not shown) regulatory regions. Also, the MM algorithm [58] (MEME method) identified the following shared motifs: [TC][CT][TC]T[CT]T[CT]T[TC]TC[TC][TC][TAC][CT]T[CT]T[CT]T[CT], with 19 and 17 motifs in *Vitis *(*Vv*) and *Arabidopsis *(*At*), respectively; G[AG]C[AC][AT][GC][AC]A[CT][CG][CA]A[CG], with 7 (*Vv*) and 2 (*At*); and C[AT]CAT[CT]TC[TC][CA][AC], with 9 (*Vv*) and 3 (*At*). The first (and more abundant) motif corresponds to (GAGA)n putative regulatory elements, which are the binding site for BASIC PENTACYSTEINE1 (BPC1), a regulator of the homeotic *Arabidopsis thaliana *gene *AGL11*, which controls ovule identity [39]. BPC1 induces conformational changes by cooperative binding to purine-rich elements (GAGAn) present in the *AGL11 *regulatory sequence [39]. Interestingly, these purine-rich repeats are abundant in the putative regulatory region of *VvAGL11*: at least six (GAGA)n were identified upstream of the TATA-box and three in the 5'UTR intron. The closest (GAGA)n repeats to the TATA-box correspond to three SSR markers segregating in the RS × S progeny, VMC7F2, VvB18B20 and p3_VvAGL11 (Figure 2 and Additional file 7). In this experimental population, p3_VvAGL11 and VMC7F2 explain up to 78% of the phenotypic variation in seedless, which make them very good candidates for being the main regulatory elements involved in the expression of the final seedless phenotype. In a selection of 146 genotypes derived from crosses of seedless × seedless varieties within our breeding program, p3_VvAGL11 yielded the highest Kruskall-Wallis value (up to 66%).

The proportion of phenotypic variation in seedlessness explained by *VvAGL11 *is huge, much greater than the estimated effect of other genes identified as QTLs from cultivated plants, like (for instance) *ovate*, which controls fruit shape in tomato (67%), and *Se1*, which controls flowering time in rice (67%) [59,60]. As discussed above, even though this result is quite consistent with previous results using similar experimental designs [4,9,10,15,18], it must be taken carefully as some degree of distortion and/or overestimation of effects could exist due to the small size of our (and other) progeny sets, genotyping and phenotyping errors and recombination slippage events in the regulatory region of *VvAGL11*. Further analyses should be performed with larger experimental designs or by transgenic assays manipulating gene expression.

The mutations identified in regulatory elements of the seedless allele of *VvAGL11 *explained a slightly higher degree of phenotypic variation than those identified in the coding region (up to 13%, 6% and 13% more in 2007, 2009 and 2010, respectively), suggesting that the seedless phenotype might be genetically controlled by this regulatory region. Transcriptional analyses performed in contrasting phenotypes as well as in homozygous seeded and seedless genotypes revealed that in seedless genotypes, the expression of *VvAGL11 *was abolished during the period of rapid seed and berry growth after berry set. As expected, in heterozygous genotypes like Sultanina or Ruby Seedless, its expression was half that observed in homozygous seeded genotypes. Together, the genetic and transcriptional evidence suggest that seedlessness in table grapes might be due to misexpression of *VvAGL11 *caused by INDELs in its regulatory elements.

Defined by intragenic marker p3_VvAGL11, the seedless allele (198 bp) exerts a partially dominant effect over the seeded alleles (188 and 192 bp): most of the heterozygous genotypes are seedless. The C-domain in the coding sequence has been described as the less conserved domain between the MADS-box family members [40]. However, each of the major MIKC sub-families possesses short, highly conserved motifs [61,62] whose specific function remains unknown [63]. The C-domain has also been reported to be involved in the mediation of higher-order interactions among MADS protein dimers [42,64], in transcriptional activation [42,65], and in post-translational modifications [66]. A non-silent mutation identified in one of these conserved motifs of VvAGL11 that did not by itself explain the seedless phenotype might be responsible for a structural change in the C-domain making the mutant transcription factor barely expressed during the initial stages of seed development and therefore dominant over its wild-type alleles.

Altogether, these results are partially agree with the model proposed by Bouquet and Danglot [14] and Lahogue et al. [18] for the control of seed development, where a single dominant locus, *SDI*, codes for a major regulatory gene. The three remaining loci that interact with *SDI*, according to the proposed model, were not identified with the current experimental design.

### Gene-assisted selection

In a perennial species such as grapevine, markers that allow individuals not carrying the favorable allele for the most desirable given trait to be discarded before planting in the field for further evaluation are invaluable. This is especially true for phenotypes that can only be screened in adult plants such as those affecting berries.

By identifying several interesting intragenic polymorphisms between seeded and seedless genotypes in the *VvAGL11 *regulatory region (p1_VvAGL11, p2_VvAGL11 and p3_VvAGL11), our study provides four new intragenic markers in a candidate gene for seedlessness for breeding purposes. These intragenic markers displayed different relative efficiencies measured as the phenotypic variation explained by the marker and based on their efficiency to select positively seedless genotypes or negatively seeded genotypes. The SSR marker VMC7F2, already described as the closest marker to the *SDI *locus [4,9,16], was confirmed as one of the best markers for progeny screening. Association analysis performed in the RS × S experimental progeny and over a population derived from several other seedless × seedless crosses revealed p3_VvAGL11 as the most reliable marker for breeding purposes over three different seasons and across different genetic backgrounds.

The two most interesting markers identified in our work or former studies (p3_VvAGL11 and VMC7F2) need to be tested for their robustness in larger genetic backgrounds segregating for seedlessness. Lahogue et al. [18] developed the SCAR marker SCC8, which is tightly linked to the *SDI *locus; however, SCC8 was not useful in all the evaluated progeny [13,18] or in the RS × S experimental population (not shown), as it often amplifies a null allele [13]. In a controlled population derived from Dominga × Autumn Seedless, Cabezas et al. [9] identified SSR markers closely linked to the *SDI *locus (VMC7F2 and VMC6F11), and these markers results in 4% to 6% false positive identifications (seeded hybrids identified as seedless) and in 11% to 13% false negatives. In the experimental population analysed in the present work, the use of marker p3_VvAGL11 for the selection of homozygous genotypes resulted in 0% false positives (Figure 4), while VMC2F2 yielded 5% false positives (data not shown). Haplotype analysis, defined either by combinations marker pairs or by all the intragenic markers for *VvAGL11 *(p1, p2, p3_VvAGL11 and VMC7F2) failed to improve the efficiency achieved by p3_VvAGL11 alone in our experimental population; any combination not only gave the same number of selected true seedless phenotypes but also increased the number of seeded phenotypes identified by mistake as true seedless (not shown).

## Conclusions

*VvAGL11 *belongs to the D-lineage of MADS-box genes that control ovule identity. A better understanding of its function would benefit other crops, as its function seems to be conserved across the plant species already studied (*A. thaliana*, *Petunia*...). However, its function in grapevine remains to be proven by genetic transformation of seeded cultivars. Whether its role in seedlessness is confirmed or not, *VvAGL11 *has proven to be a very useful marker for assisted selection of seedless grapevine.

## Methods

### Plant material

For QTL mapping experiments, full sib progeny were obtained via embryo rescue [12] from a cross between Ruby Seedless and Sultanina (RS × S [16]; N = 139); seedlings from this progeny were grown on their own roots or over Sultanina rootstock as a replicate. For validation purposes, 146 mature seedlings derived from 14 different crosses between 11 seedless varieties were used for genotyping and phenotyping experiments. All genotypes were grown at La Platina Experimental Station of the Instituto de Investigaciones Agropecuarias, Santiago, Chile. A core collection (N = 21) was also used to test the association between the identified polymorphisms and traits, and this collection contains a representative sample of diversity in cultivated *Vitis vinifera *L. and in different *Vitis *species and genera [67] (Additional file 10). The core collection and genotypes of the *Vitis *genus are held by INRA Montpellier, France, at the domain of Vassal, F-34340 Marseillan (http://www.montpellier.inra.fr/vassal). The core collection is a sub-sample of 48 varieties selected based on their genotypes for 20 SSR markers using the M-strategy. This core collection, highly non-redundant and highly diverse, represents 83% of the total SSR diversity [67] from the world largest germplasm collection of cultivated *Vitis vinifera*, 3,900 accessions corresponding to 2,262 unique genotypes (Laucou et al. cited in [67]). In all cases, genomic DNA was extracted according to Lodhi et al. [68] from 100 mg of young immature leaves (not fully expanded) collected two weeks after bud-break and kept at -80°C or lyophilized until DNA extraction.

### Phenotypic evaluations

Seedlessness can be dissected into three main sub-traits, seed fresh weight, seed dry weight and seed number [9,10]. In this work, seedlessness was analysed as seed fresh weight because no significant differences were found between fresh and dry weight in a preliminary analysis [16] and because seed number analysis is subject to bias due to the subjectivity of determining and differentiating true seeds from large rudiments, or rudiment traces from ovule traces.

Phenotypic data were recorded using an improved protocol from 115, 126 and 122 mature individuals from the 2007, 2009 and 2010 seasons, respectively, which are 17, 28 and 24 more than in the former QTL detection study with the same progeny [16]. Briefly, both berry weight (g) (BW) and seed fresh weight (g) (SFW) were scored at the ripening stage (17 ° Brix). For BW and SFW, 300 berries and seeds from 150 berries, respectively, were randomly sampled and weighed in three different clusters of each genotype. Quantitative analyses were performed of the mean BW per genotype and the mean SFW per berry and per genotype. For validation purposes, the same phenotyping strategy was used to analyze a population (n = 146) issued from 14 different crosses between common seedless varieties: Sultanina × Ruby Seedless (n = 30), Beauty Seedless × Crimson Seedless (n = 19), Red Seedless × Flame Seedless (n = 5), Ruby Seedless × Perlette (n = 7), Sultanina × Black Seedless (n = 9), Flame × Black Seedless (n = 10), Ruby Seedless × Superior Seedless (n = 9), Ruby Seedless × Dawn Seedless (n = 28), Flame Seedless × Perlette (n = 3), Flame Seedless × Beauty Seedless (n = 4), Ruby Seedless × Beauty Seedless (n = 4), Red Seedless × Dawn Seedless (n = 7), Sultanina × Dawn Seedless (n = 7) and Sultanina × Superior Seedless (n = 4). Association analysis was performed by one-way ANOVA, significative differences were tested at *P *< 0.05 by Fisher's least significant difference procedure.

The dominance effect *d *was calculated according to Acquaah [69] as follows: *d = Mab -[(Maa + Mbb)/2] *where *M *is the phenotypic mean of the genotypes *(aa *seedless homozygous genotypes, *bb *seeded homozygous genotypes and *ab *heterozygous genotypes); if *d *< 0, the *a *allele presents incomplete dominance (partial dominance) over the *b *allele.

### SSR and *VvAGL11 *genotyping

To reduce the confidence interval of the major seedlessness QTL identified previously on chromosome 18, a total of 13 publicly available SSR primer pairs were selected according to the Costantini et al. [70] strategy and based on existing reference maps [43,71,72]. Fifteen new SSR markers were developed from Cabernet-Sauvignon BAC End Sequences (BES) [73] or from the currently available assemblies of the grapevine genome sequencing project [1,32] using the SSRIT software [74]; the developed SSR markers are described in Additional file 3. The SSR search was directed to the QTL-containing region or to poorly integrated regions between the physical and genetic maps. As an example, in the region of the SSR marker VMC7F2, both BES of the BAC contig n°1821 of the Cabernet-Sauvignon physical map (http://urgi.versailles.inra.fr/cmap) and sequences from the 6X genome assembly [1,32] matching these BES were used, comparisons between BES and sequences from the genome assembly were performed by BLASTn [75]. Primers were designed using the Primer3 software [76], and they were used for BAC anchoring experiments according to Lamoureux et al. [73] and for genetic mapping experiments.

*VvAGL11 *was identified as the most evident positional candidate gene in the defined confidence interval for the major seedlessness QTL on chromosome 18. As soon as the 8.4x annotated grapevine genome sequence was available [1,32], its annotation was used to confirm its true orthologous relationship by a reciprocal best match procedure as described in [1]. Gene models and predicted coding sequences from the automatic annotation of the grapevine genome sequence [1] that were identified in QTL regions were carefully checked using the available resources. In particular, we checked the alignment of *Vitis *ESTs from public databases (NCBI) or from a private EST database [77] that holds 18,366 ESTs derived from libraries of different floral and berry developmental stages in cvs. Sultanina and Carmenère.

General genotyping PCR amplifications were done in a 10-μL reaction mixture containing 0.25 μM each primer, 0.25 mM each dNTP, 1.6 mM MgCl_2_, 0.25 U Taq polymerase, 25 ng of template DNA, 0.2 mM Red Cresol and 12% sucrose. An Amp^® ^PCR system 9700 (PE Applied Biosystems) was programmed as follows for PCR amplification: 30 sec at 95°C, annealing (30 sec at 58°C), and extension (30 sec at 72°C) for 35 cycles, followed by a fill-in step of 4 min at 72°C. SSRs were resolved by denaturing acrylamide gel electrophoresis according to Creste et al. [78] with some modifications: a 6% acrylamide solution 37.5:1 (acrylamide:bisacrylamide) with 7 M urea and 3.75% glycerol was used. SSCPs were resolved in MDE (FMC BioProducts Inc) gels according to Martins-Lopes et al. [79] or in native 8% acrylamide (37.5:1) and 5% glycerol gels. After electrophoresis in native, denaturing or MDE gels, the amplified fragments were revealed by silver staining according to Creste et al. [78]. For *VvAGL11 *intragenic markers (Additional file 3) the annealing temperature was set to 64°C, the rest was as above. For p3_VvAGL11 specifically, PCR products labeled with PET dye were resolved by capillary electrophoresis according to standard procedures recommended for the ABI 3130xl Genetic Analyzer; the other parameters used were as described above.

### Genetic map construction for LG18

In heterozygous plant species like *Vitis*, the various marker pairs segregation type greatly differ in their accuracy for estimation of recombination frequency with regard to the power for detecting linkage [80]. After markers have been assigned to linkage groups, conflicting information with respect to the marker order is often provided by the different pairwise recombination frequency estimates. This can be due to missing marker data, but also to random estimation errors in the recombination frequency inherent to the marker configurations [80]. To reduce such problems, we built linkage group 18 using co-dominant markers only and the fixed order option based on the available genomic sequence [1,32]. The double pseudo-testcross strategy [81] and JoinMap 3.0 software [82] were used to automatically determine the phases and to build the genetic map. Markers with high segregation distortion, unexpected χ2 test results or null alleles (a_ × ab; ab × a_) that cannot be handled by JoinMap 3.0 were discarded or scored as dominant markers. The LOD score and recombination threshold for the determination of linkage groups were, respectively set at 3.5 and 0.4. Markers within the resulting groups were ordered relative to each other by automatic multipoint analyses using the default values of JoinMap 3.0 (mapping threshold LOD > 1, REC < 0.4). Parental maps were constructed as two cross-pollinated populations. A consensus map was constructed using the parameters for a cross-pollinated derived population and the integrate map function of JoinMap 3.0. Recombination units were transformed into genetic distances using the Kosambi function [83]. The linkage group was numbered according to the recommendation of the IGGP [84].

### QTL analysis

Phenotypic data were submitted to basic statistics and normality tests with Minitab 15 software (Minitab Inc). Data were normalized with the Johnson transformation included in Minitab 15. QTL detection and analyses by interval mapping [85] were performed separately for both parental and consensus framework maps using MapQTL 4.0 [86] and the normalized data for BW and SFW. To establish the confidence of a putative QTL, the following strategy was undertaken. For each putative QTL, the closest markers to the peak of the LOD profile were tested using the Automatic Cofactor Selection procedure. Markers accepted as co-factors where then used to perform a Multiple QTL Mapping test and to determine the total phenotypic variation explained by these markers. In parallel, a Permutation Test (1,000 permutations, genome-wise and chromosome-wise type error rate of 0.05) was used to establish the threshold level at which a QTL was declared significant or suggestive [87]. QTLs were established as significant when the detected LOD was higher than the threshold LOD for a genome-wise type error. One-LOD and two-LOD support confidence intervals were constructed for each QTL [85]. Associations between alleles of intragenic *VvAGL11 *markers and phenotypes were further assessed with the non-parametric Kruskal-Wallis (KW) rank-sum test using the non-normalized phenotypic data.

### Sequence analysis

*VvAGL11 *has an expected size near 10 kb comprising the putative regulatory and coding regions. Besides, it is in heterozygous state in both parental genotypes, which makes amplification, cloning and sequence assembly difficult. Therefore, we decided to isolate the regulatory sequence from DNA and the coding sequence from cDNA, both isolated in homozygous genotypes (defined by their genotype at the VMC7F2 marker).

Primers were designed with the Primer3Plus web interface [88] using the sequencing option and the PN40024 genome sequence as the template (Additional file 14). PCR products were amplified in the same conditions as described for the genotyping procedure, and the amplicons were purified with a QIAEX II^® ^Gel Extraction Kit (QIAGEN) and cloned into pGEM-T-Easy^® ^(Promega) for sequencing. Sequence trimming and contig assembly were performed with Geneious^® ^[89]. The partially sequenced regulatory region corresponds to ~1.5 kb upstream and ~1.4 kb downstream of the TATA box, and the 1.4 kb region includes the 5'UTR intron. Regulatory sequence analysis of *VvAGL11 *from PN40024 and from both Sultanina-derived alleles was performed using the PLACE database [37]. The search for conserved motifs in the regulatory region between *Vitis *and *Arabidopsis *was performed by the MEME method [58].

The coding region was cloned and sequenced from RNA isolated from the same genotypes as described above in three different developmental stages (I, J and K according to Baggiolini [90]). Total RNA was extracted with the FavorPrep Total RNA Mini Kit for Woody Plants^® ^(FAVORGEN), the mRNA was purified with Dynabeads^® ^Oligo(dT) (INVITROGEN) and cDNA was amplified with SuperScript III RT^® ^(INVITROGEN). The oligos for *VvAGL11 *CDS isolation are 5'-ATGGGGAGAGGAAAGATCGA-3' and 5'-TACCCGAGATGGAGGACCTT-3', and the PCR conditions were the same as described above. Bands of the expected size (671 bp) were cut from agarose gels and purified and cloned as described above; four clones from each genotype were sequenced.

### Genetic analysis of *VvAGL11 *polymorphisms

Four intragenic markers were developed located in the regulatory (3) and coding (1) regions: p1, p2 and p3_VvAGL11 and e7_VvAGL11, respectively (Additional file 3 and Figure 2B). The p1, p2 and p3 markers are SSR-like and e7 is an SSCP marker. e7_VvAGL11 amplicons from two representative seedlings of each genotype (four genotypes 1:1:1:1, ee, ef, eg, fg) plus both parental genotypes (ef and eg for RS and S, respectively) were cloned into pGEM-T-Easy^® ^(Promega). Clones showing different inserts (alleles) were chosen by SSCP analysis for sequencing using transformed colonies directly as PCR templates. The region containing the marker p3_VvAGL11 and defined as the putative minimal promoter was amplified using template DNA from a seeded genotype that presented a new second allele using AccuPrime Pfx DNA polymerase (Invitrogen) and cloned into pENTR/D-TOPO (Invitrogen). The oligos used to isolate this region are 5'-caccTTGTGGCCTTGAAGAAA-3' and 5'-CACAATGGAGAGATGTGAGACG-3', and the manufacturer's conditions were followed for the PCR, purification and ligation reactions.

### Real-time quantitative PCR (qPCR) assays

The transcript abundance of *VvAGL11 *was evaluated in the four genotypes of the RS × S progeny described above for sequence characterization: both heterozygous seedless parents of the progeny (Ruby Seedless and Sultanina), and an unrelated seeded common table grape genotype, Red Globe. Expression analysis was performed at three developmental stages of fruit development (pre-bloom, I; bloom, J; and fruit set with berries showing 5-10 mm equatorial diameter, K) according to Baggiolini [90]). Three biological samples where independently analyzed for each genotype × stage combination.

qPCR was performed with the LightCycler^® ^(Roche Diagnostics) real-time PCR system using SYBR Green^® ^as the fluorescent dye to measure DNA amplicons derived from mRNA. A 100-ng aliquot of mRNA was used as the template for reverse transcription reactions to synthesize single-stranded cDNA using the SuperScript III^® ^system and oligo(dT) primers (INVITROGEN) according to standard procedures. Gene-specific primers were designed with Primer3 [76] considering exon-exon junctions. For *VvAGL11*, the oligos are 5'-GCAGAAGTTGCCCTCATCGT-3' and 5'-AAGCCAAGGAATCACCCATT-3'; for the internal reference gene EF1-α (GSVIVT00024496001-8.4x) the oligos are 5'-AGGATGGACAAACCCGTGAG-3' and 5'-AAGCCAGAGATGGGGACAAA-3', and the amplicons have a predicted size of 232 bp and 202 bp, respectively. For each gene, a calibration curve was constructed by measuring the fluorescence of four serial dilutions (10^1^-10^-2 ^pg ul^-1^) of an RT-PCR product obtained with the same oligos and cDNA from PN40024 as the template to estimate copy numbers in total cDNA.

The amplification reaction was carried out in a total volume of 20 μl containing 1 pmol of each primer, 1.5 mM MgCl2, 1 μl of LightCycler^® ^DNA Master SYBR Green I (containing 1.25 U of Taq polymerase, 10× Taq buffer (500 mM KCl, 100 mM TRIS-HCl, pH 8.3), dNTPs each at 2 mM, 10× SYBR Green I; (Roche Diagnostics) and 100 ng of cDNA prepared as described above.

The thermal conditions for qPCR were as follows: denaturation at 95°C for 10 min, followed by 35 three-step cycles of template denaturation at 95°C with a 2 s hold, primer annealing at 60°C for 10 s, and extension at 72°C for 20 s. Fluorescence data were collected after each extension step. Melting curve analyses were performed by heating the template at 95°C with a 0 s hold, then cooling to 60°C with a 15 s hold, and finally increasing the temperature to 95°C with a 0.1°C s^-1 ^temperature transition rate while continuously monitoring the fluorescence. All other phases were performed with a 20°C s^-1 ^transition rate. Fluorescence was analyzed using LightCycler^® ^Analysis Software. The crossing point for each reaction was determined using the second derivative maximum algorithm and manual baseline adjustment. In all cases, the melting curves were checked for single peaks, and the amplification product sizes were confirmed in agarose gels to ensure the absence of non-specific PCR products. Duplicate qPCR experiments were performed for each sample. If a statistical difference was found between the two replicates, one to two additional replicates were added. The expression values were normalized against EF1-α. To test whether EF1-α behaved as a housekeeping gene in the analyzed samples, cDNA samples from the three stages of berry development (I, J and K) were analyzed comparing EF1-α and actin as a control transcript (GSVIVT00034893001, primers 5'-GCTGGATTCTGGTGATGGTG-3' and 5'-CCAATGAGAGATGGCTGGAA-3', 348 bp product size). For each cDNA, the transcript abundances of EF1-α and actin were analyzed by qPCR and the ratios of the control transcript to the endogenous EF1-α transcript were calculated. The results indicated that the abundance of EF1-α mRNA remained stable between samples (data not shown). qPCR data normalized with the LOG10 function and subjected to statistical analyses of variance and treatment means were separated using Tukey's Post-hoc test at *P *= 0.05 with Prism^® ^v4.0 (GRAPHPAD).

## Authors' contributions

NM conceived the experimental design; performed the genotyping, phenotyping, sequence and database analyses; performed marker design and statistical, linkage and QTL analyses; and designed and drafted the manuscript. BS and MG contributed equally to the genotyping and phenotyping of the progeny. XC performed the qPCR experiments, phenotypic evaluations over Sultanina rootstocks and statistical analyses. MAM and RR performed the directional cloning of the putative minimal promoter and coding sequences and assisted in all the molecular techniques. LLC and JMB developed the core collection, checked the associated phenotypes and discussed and edited the manuscript. CH performed the sequence analysis of *VvAGL11 *in the Core Collection. AFAB coordinated and followed the experiments for the integrative mapping, participated in the analysis of the results and discussions and in the editing of the manuscript. PH participated in the initial design of the project, discussions and editing the manuscript. All authors have read and approved the final version of the manuscript.

## Supplementary Material

Additional file 1 **Phenotypic distributions for mean seed fresh weight (A) and mean berry weight (B) in the studied full sib family for 2007, 2009 and 2010 seasons**. Seedlings evaluated in 2007 were grown on their own roots and seedlings evaluated in 2009 and 2010 were grafted over Sultanina rootstocks. Click here for file

Additional file 2 **Correlation between seed and berry weight**. Scatter plots of the full sib progeny for seed fresh weight and berry weight evaluated in 2007, 2009 and 2010 seasons. Lines represents the linear regression model between berry weight and seed fresh weight with correlation coefficients r^2 ^= 0.41, 0.44 and 0.46 for 2007, 2009 and 2010 respectively. Click here for file

Additional file 3 **New Simple Sequence Repeats (SSRs) and VvAGL11 intragenic markers mapped in linkage group 18**. Name of the marker (Marker ID); accession number; Forward and Reverse primer sequences, amplicon size (Size) and PCR conditions (annealing temperature and magnesium concentration). Loci size is indicated by default for the Pinot Noir PN40024 reference genome. One or two asterisks indicate the size of the seedless and seeded allele for *VvAGL11 *intragenic markers respectively; size was determined by capillary electrophoresis considering adenine overhangs at both 3' ends. Click here for file

Additional file 4 **Number and segregation type of co-dominant markers used in the linkage analysis of chromosome 18**. Segregation type corresponds to Cross-Pollinated nomenclature for Joinmap 3.0. Click here for file

Additional file 5 **Gene models contained in the 92.038 kb confidence interval of QTLs for seedlessness and berry weight**. Gene ID according to the grapevine genome browser; accession number of the SwissProt best match and inferred possible function (Best match description) and position on the genome sequence in bp (Position). Click here for file

Additional file 6 **Phylogram of the *AGAMOUS *family generated by ClustalW**. The analysis includes sequences from C and D-class gene families. The Jukes-Cantor model was used for determination of genetic distance and the tree was built with UPGMA. Sequences have the following origin: *O. sativa, OsMADS13 *[Swiss-Prot:Q2QW53]; *Lilium longiflorum*, *LMADS2*, [GenBank:AAS01766]; *A. thaliana, AG *[GenBank:NP_567569], *SHP1 *[GenBank:NP_191437.1], *SHP2 *[GenBank:NP_850377.1] and *AGL11 *[GenBank:NP_192734.1]; *P. hybrida, FBP7 *[GenBank:CAA57311.1] and *FBP11 *[GenBank:CAA57445.1]; *V. vinifera, VvMADS5 *[GenBank:AAM21345.1], Sultanina Seedless and Seeded-derived alleles of *VvAGL11 *[GenBank:CAO1637]; *Lilium longiflorum*, *LMADS2 *[GenBank:AAS01766]; *Gossypium hirsutum *[GenBank:AAN15183]; *Cucumis sativus *[GenBank:AAC08529]; *Lotus corniculatus *[GenBank:AAX13306], *Malus × domestica *[GenBank:CAA04324]; *Prunus persica *[GenBank:ABQ85556] and *Prunus dulcis *[GenBank:AAY30856]. Click here for file

Additional file 7 **Predicted cis-regulatory elements that differ between seeded (pSEEDED) and seedless (pSEEDLESS) putative minimal regulatory region of *VvAGL11***. Both sequences were aligned on the genome reference sequence (pPN40024). SNPs and INDELs are signalled by coloured bases or sequence gaps. Yellow and blue segments represent 5'UTRs and TATA-box, putative cis-regulatory elements identified by PLACE database are indicated with brown segments with their respective accession number (last three digits). Red segments represent the polymorphic markers mapped in the RS × S experimental progeny. Click here for file

Additional file 8 **Predicted cis-regulatory elements identified by PLACE database that differ between the seeded and seedless putative minimun regulatory region (430 bp upstream the transcription start site) and the first intron (1.4 kb of the 5'UTR intron)**. Seeded and Seedless sequenced Sultanina-derived alleles were aligned and scanned for motifs by PLACE database. Click here for file

Additional file 9 **Transcript differences between seeded and seedless alleles derived from the RS × S progeny**. Nucleotidic and amino-acidic sequences from seedless (SEEDLESS cDNA VvAGL11) and seeded (SEEDED cDNA VvAGL11) alleles were aligned and compared against the predicted CDS from PN40024 (virtual cDNA VvAGL11). SNPs and non-silent mutations are signalled by coloured nucleotides or amino acids. Exons are represented by grey segments and size is in bp relative to the ATG. Click here for file

Additional file 10 **Nucleotide diversity of *VvAGL11 *exon 7 in a collection of *Vitis vinifera *genotypes maximizing sequence diversity and a few *Vitis *species**. Both already known seedless and seeded alleles from Ruby Seedless and Sultanina were included as well as Syrah (VvMADS5:SYH) and PN40024 (PNI). Exon 7 was obtained from a direct sequencing of PCR products using genomic DNA of the following genotypes as a template: cultivated *Vitis vinifera *such as Kishmish Chernyi (KIC), Asyl Kara (ASS), Orlovi Nokti Beli (ORL), Katta Kurgan (MAK), Araklinos (ARA), Arbois (ARB), Chardchi (CHB), Kapistoni Tetri (KAP), Médouar (MED), Mehdik (MEH), Oasis Bou Chemma 46 (OA7), Pletchistik (PLE), Tsitsa Kaprei (TIC), Tzolikoouri (TSO) and Lambrusque E (LAE), members of the *Vitis *genus such as *Vitis berlandieri *(VBE), *Vitis aestivalis *(VAE), *Vitis coignetiae *(VCO), *Vitis labrusca *(VLI) and *Vitis rupestris *(VRU), and one wild *Vitis vinifera *such as Lambrusque Sejnene 1 (LAS). Polymorphisms are signaled by colored nucleotides or amino acids. An asterisk signals seedless genotypes. Click here for file

Additional file 11 ***VvAGL11 *intragenic marker mapping and QTL analysis for seedlessness and berry size detected over three different seasons on chromosome 18**. A: Consensus genetic map of chromosome 18 based on the RS × S progeny. Green, pink and red markers correspond to SSRs developed in this study from Cabernet Sauvignon BAC End Sequence, from contig assemblies of the grapevine genome sequencing project, and from *VvAGL11 *allele sequencing, respectively. B and C: Projected seedlessness and berry size QTLs represented by coloured vertical bars and LOD (logarithm of the odds) profiles to the right of chromosome 18. Red, blue and green lines correspond to 2007, 2009 and 2010 seasons, respectively. Bar lengths are representative of their confidence interval once projected on the consensus map. Seedlessness was analyzed as seed fresh weight (SFW) and berry size as berry weight (BW). 1-LOD and 2-LOD support intervals were used for the prediction of the confidence intervals. Vertical dashed line in the LOD profile represents the LOD threshold for significant QTLs according to the permutation tests. Genetic distances are expressed in centimorgans (cM). Click here for file

Additional file 12 **Association analysis performed in a population derived from crosses of several seedless varieties**. A population (N = 146 seedlings) originating from 14 progeny derived from crosses of 11 seedless varieties (Perlette, Flame Seedless, Beauty Seedless, Dawn Seedless, Black Seedless, Melissa, Crimson, Red Seedless, Ruby Seedless, Superior Seedless and Sultanina) was genotyped with intragenic marker p3_VvAGL11, and mean seed fresh weight per berry was recorded. Allele sizes were determined by capillary electrophoresis. Association analysis was performed by one-way ANOVA, and different letters represent significant differences at *P *< 0.05 by Fisher's least significant difference procedure. Click here for file

Additional file 13 **Genetic and physical distance between markers comprised within the confidence interval**. Microsatellite repeat and segregation type, relative position in linkage map, distance between loci, position in the reference genome assembly, physical distance between loci and recombination frequency between adjacent markers is indicated. Underlined markers belong to the confidence interval from the major QTL for seedlessness reported in this work. Click here for file

Additional file 14 **Primer pairs designed to sequence the regulatory region of VvAGL11**. Oligos were designed by Primer3Plus web interface using PN40024 sequence as template. Click here for file
