# Supplementary figures and images for: Molecular, genetic and transcriptional evidence for a role of VvAGL11 in stenospermocarpic seedlessness in grapevine
Source: BMC Plant Biol. 2011 Mar 29;11:57. doi: 10.1186/1471-2229-11-57 (PMC3076230; doi:10.1186/1471-2229-11-57)

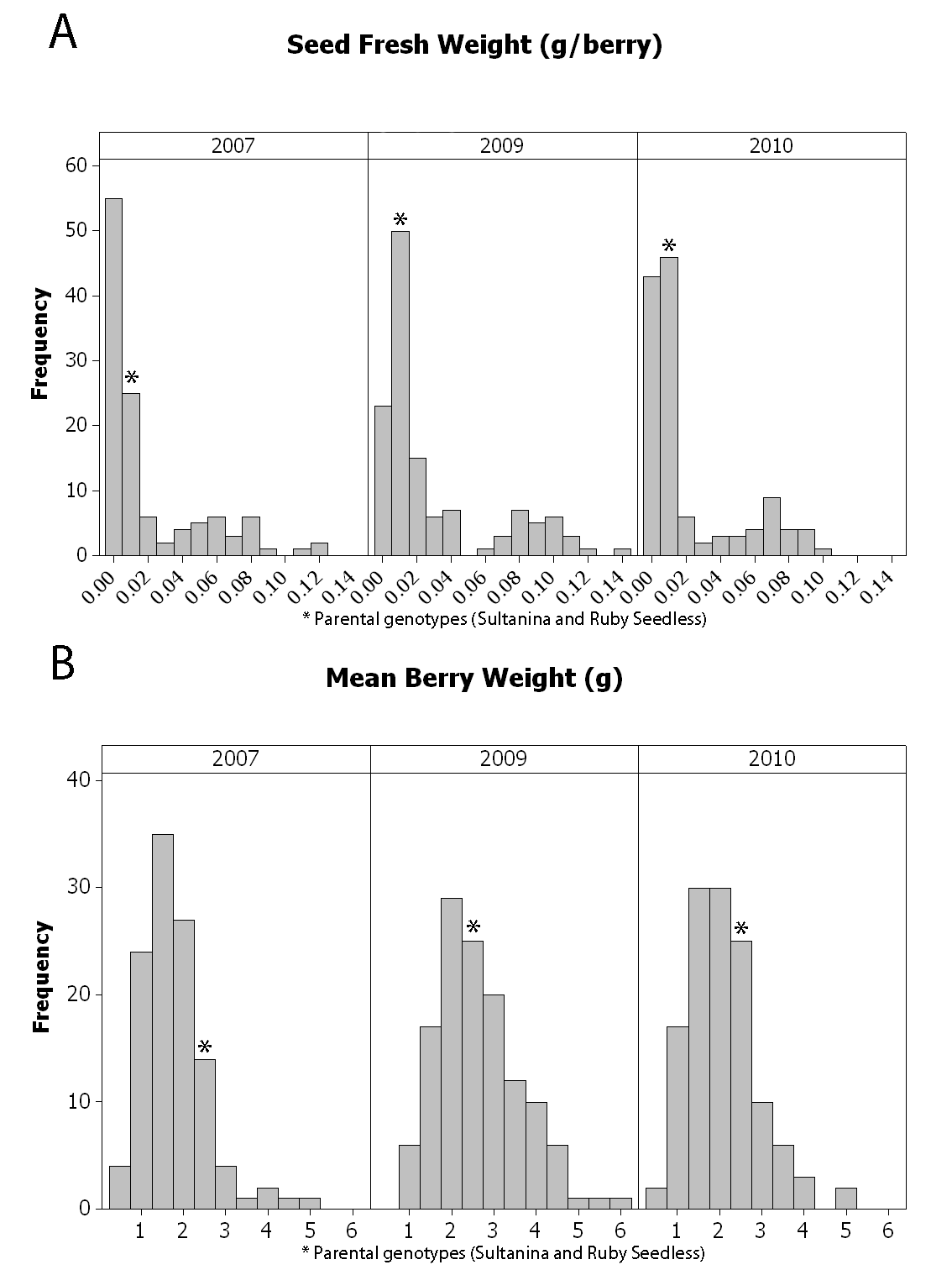

Supplement: Additional file 1 — Phenotypic distributions for mean seed fresh weight (A) and mean berry weight (B) in the studied full sib family for 2007, 2009 and 2010 seasons. Seedlings evaluated in 2007 were grown on their own roots and seedlings evaluated in 2009 and 2010 were grafted over Sultanina rootstocks. [file 1471-2229-11-57-S1.TIFF]

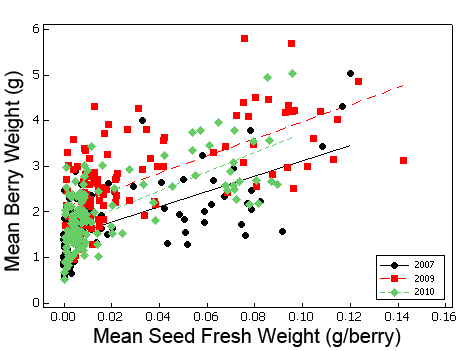

Supplement: Additional file 2 — Correlation between seed and berry weight. Scatter plots of the full sib progeny for seed fresh weight and berry weight evaluated in 2007, 2009 and 2010 seasons. Lines represents the linear regression model between berry weight and seed fresh weight with correlation coefficients r2 = 0.41, 0.44 and 0.46 for 2007, 2009 and 2010 respectively. [file 1471-2229-11-57-S2.TIFF]

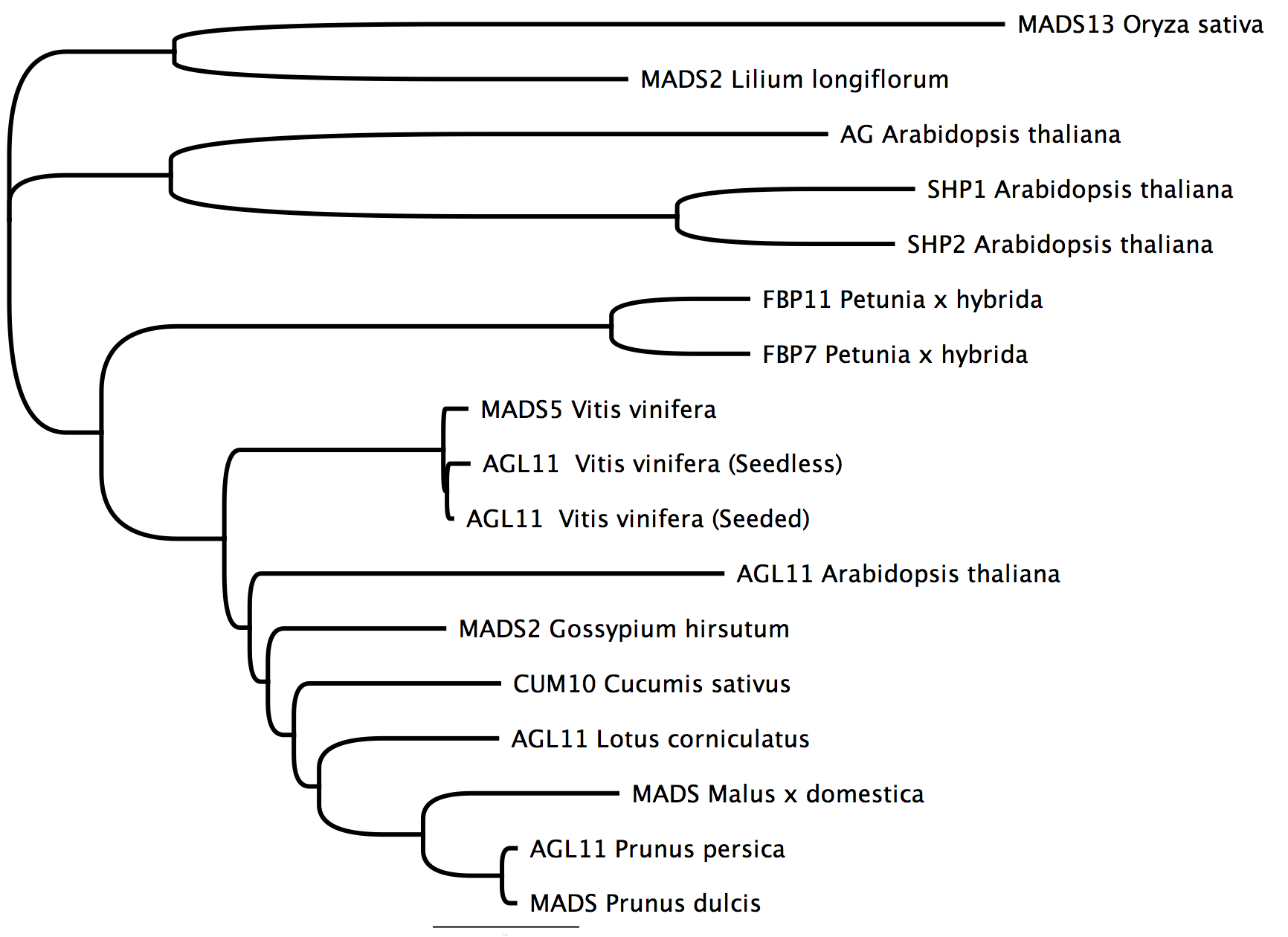

Supplement: Additional file 6 — Phylogram of the AGAMOUS family generated by ClustalW. The analysis includes sequences from C and D-class gene families. The Jukes-Cantor model was used for determination of genetic distance and the tree was built with UPGMA. Sequences have the following origin: O. sativa, OsMADS13 [Swiss-Prot:Q2QW53]; Lilium longiflorum, LMADS2, [GenBank:AAS01766]; A. thaliana, AG [GenBank:NP_567569], SHP1 [GenBank:NP_191437.1], SHP2 [GenBank:NP_850377.1] and AGL11 [GenBank:NP_192734.1]; P. hybrida, FBP7 [GenBank:CAA57311.1] and FBP11 [GenBank:CAA57445.1]; V. vinifera, VvMADS5 [GenBank:AAM21345.1], Sultanina Seedless and Seeded-derived alleles of VvAGL11 [GenBank:CAO1637]; Lilium longiflorum, LMADS2 [GenBank:AAS01766]; Gossypium hirsutum [GenBank:AAN15183]; Cucumis sativus [GenBank:AAC08529]; Lotus corniculatus [GenBank:AAX13306], Malus × domestica [GenBank:CAA04324]; Prunus persica [GenBank:ABQ85556] and Prunus dulcis [GenBank:AAY30856]. [file 1471-2229-11-57-S6.PNG]

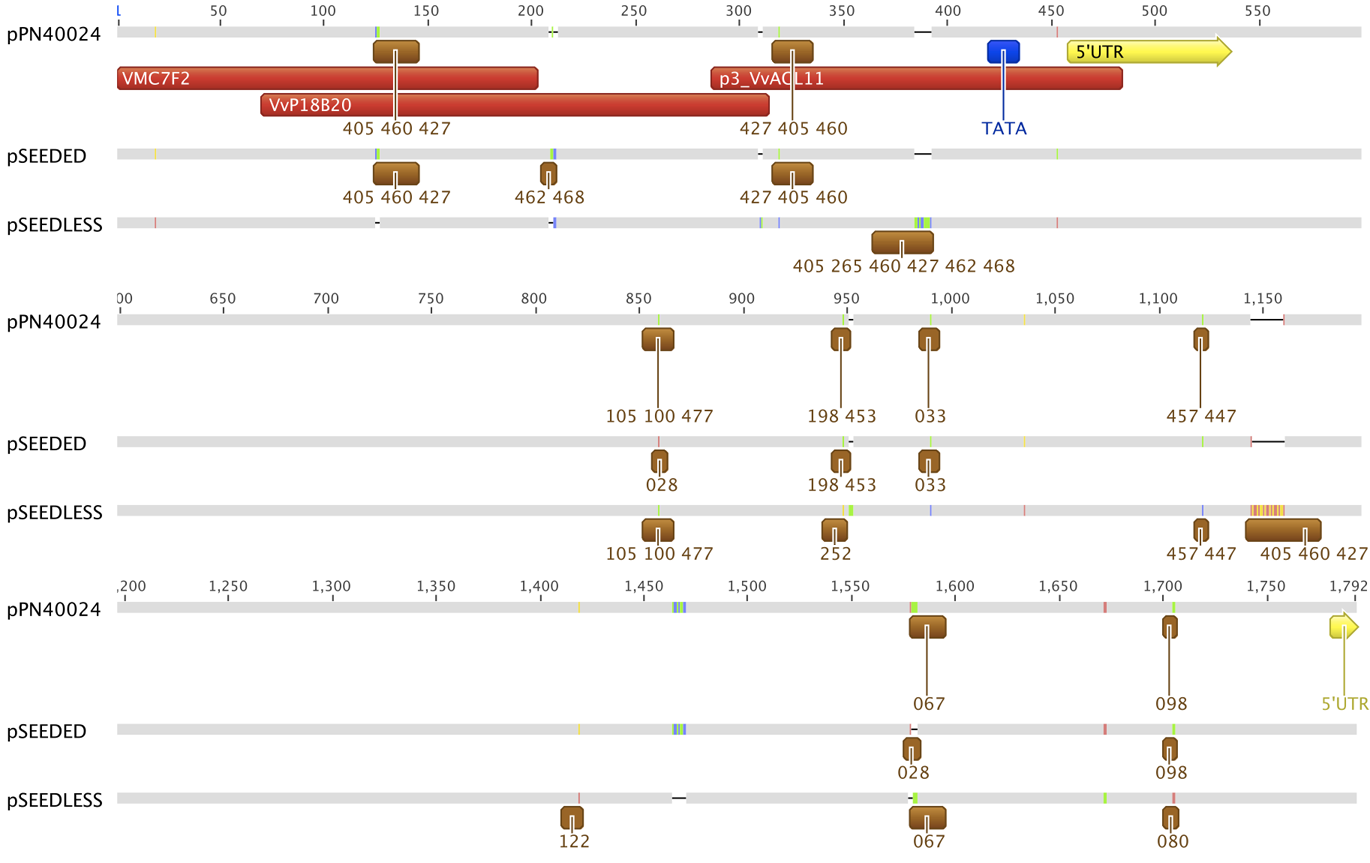

Supplement: Additional file 7 — Predicted cis-regulatory elements that differ between seeded (pSEEDED) and seedless (pSEEDLESS) putative minimal regulatory region of VvAGL11. Both sequences were aligned on the genome reference sequence (pPN40024). SNPs and INDELs are signalled by coloured bases or sequence gaps. Yellow and blue segments represent 5'UTRs and TATA-box, putative cis-regulatory elements identified by PLACE database are indicated with brown segments with their respective accession number (last three digits). Red segments represent the polymorphic markers mapped in the RS × S experimental progeny. [file 1471-2229-11-57-S7.TIFF]

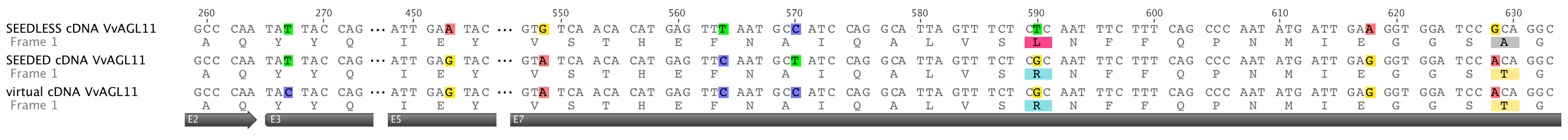

Supplement: Additional file 9 — Transcript differences between seeded and seedless alleles derived from the RS × S progeny. Nucleotidic and amino-acidic sequences from seedless (SEEDLESS cDNA VvAGL11) and seeded (SEEDED cDNA VvAGL11) alleles were aligned and compared against the predicted CDS from PN40024 (virtual cDNA VvAGL11). SNPs and non-silent mutations are signalled by coloured nucleotides or amino acids. Exons are represented by grey segments and size is in bp relative to the ATG. [file 1471-2229-11-57-S9.PNG]

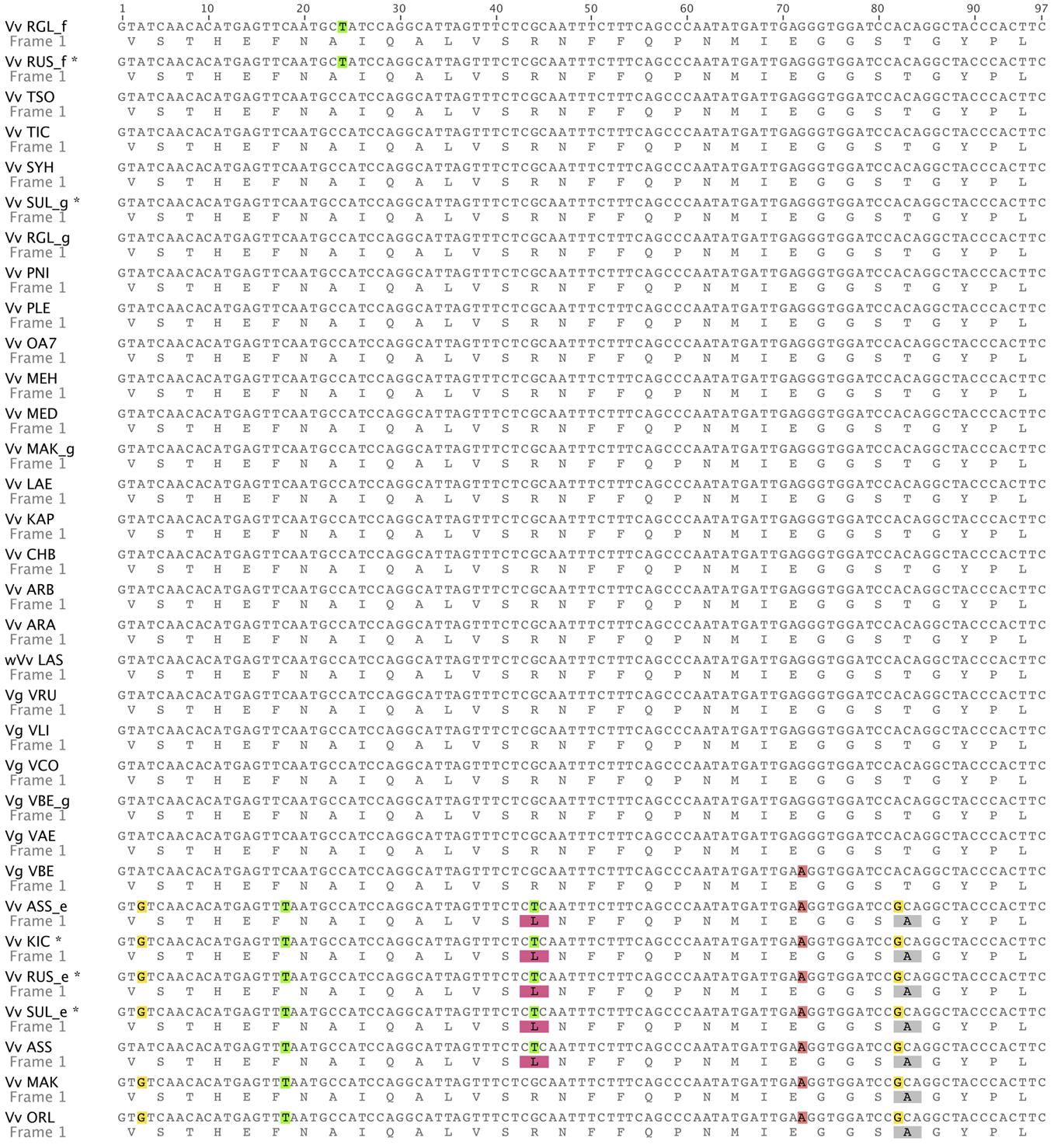

Supplement: Additional file 10 — Nucleotide diversity of VvAGL11 exon 7 in a collection of Vitis vinifera genotypes maximizing sequence diversity and a few Vitis species. Both already known seedless and seeded alleles from Ruby Seedless and Sultanina were included as well as Syrah (VvMADS5:SYH) and PN40024 (PNI). Exon 7 was obtained from a direct sequencing of PCR products using genomic DNA of the following genotypes as a template: cultivated Vitis vinifera such as Kishmish Chernyi (KIC), Asyl Kara (ASS), Orlovi Nokti Beli (ORL), Katta Kurgan (MAK), Araklinos (ARA), Arbois (ARB), Chardchi (CHB), Kapistoni Tetri (KAP), Médouar (MED), Mehdik (MEH), Oasis Bou Chemma 46 (OA7), Pletchistik (PLE), Tsitsa Kaprei (TIC), Tzolikoouri (TSO) and Lambrusque E (LAE), members of the Vitis genus such as Vitis berlandieri (VBE), Vitis aestivalis (VAE), Vitis coignetiae (VCO), Vitis labrusca (VLI) and Vitis rupestris (VRU), and one wild Vitis vinifera such as Lambrusque Sejnene 1 (LAS). Polymorphisms are signaled by colored nucleotides or amino acids. An asterisk signals seedless genotypes. [file 1471-2229-11-57-S10.TIFF]

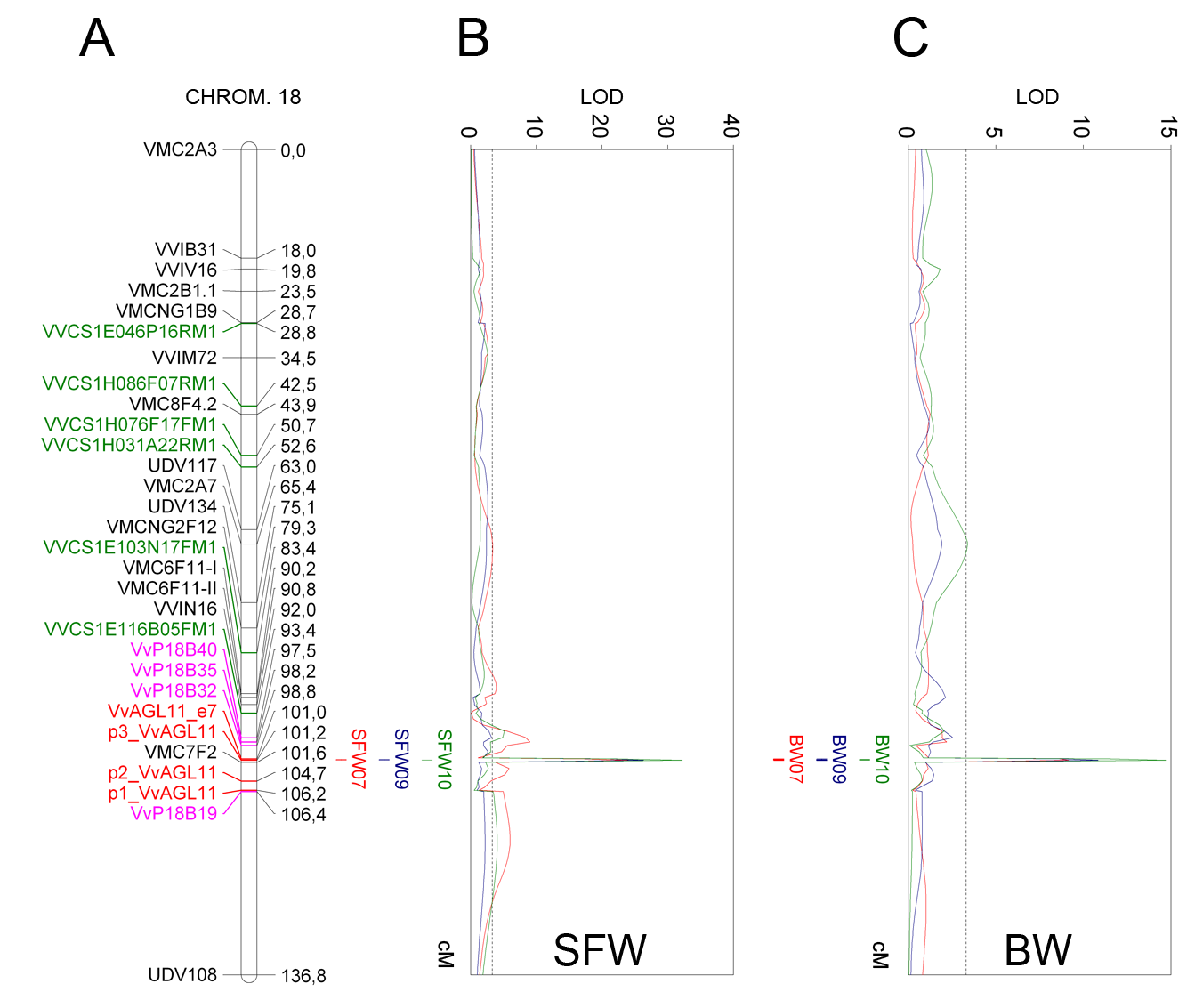

Supplement: Additional file 11 — VvAGL11 intragenic marker mapping and QTL analysis for seedlessness and berry size detected over three different seasons on chromosome 18. A: Consensus genetic map of chromosome 18 based on the RS × S progeny. Green, pink and red markers correspond to SSRs developed in this study from Cabernet Sauvignon BAC End Sequence, from contig assemblies of the grapevine genome sequencing project, and from VvAGL11 allele sequencing, respectively. B and C: Projected seedlessness and berry size QTLs represented by coloured vertical bars and LOD (logarithm of the odds) profiles to the right of chromosome 18. Red, blue and green lines correspond to 2007, 2009 and 2010 seasons, respectively. Bar lengths are representative of their confidence interval once projected on the consensus map. Seedlessness was analyzed as seed fresh weight (SFW) and berry size as berry weight (BW). 1-LOD and 2-LOD support intervals were used for the prediction of the confidence intervals. Vertical dashed line in the LOD profile represents the LOD threshold for significant QTLs according to the permutation tests. Genetic distances are expressed in centimorgans (cM). [file 1471-2229-11-57-S11.TIFF]
